# Undergraduate students’ norms for the Chinese version of the symptom check-List-90-R (SCL-90-R)

**DOI:** 10.1186/s12889-020-09689-z

**Published:** 2020-10-21

**Authors:** Yulan Yu, Chonghua Wan, Xudong Zhao, E. Scott Huebner, Jianfeng Tan, Chuanzhi Xu, Jingjing Zhang

**Affiliations:** 1 Department of Psychology Research Center for Quality of Life and Applied Psychology, Guangdong Medical University, Dongguan, 523808 China; 2 School of Humanities and Management, Research Center for Quality of Life and Applied Psychology, Guangdong Medical University, Dongguan, 523808 China; 3 Institute of Psychosomatic Medicine, The East Translational Medicine Platform, Tongji University, Shanghai, 200092 China; 4 Department of Psychology, University of South Carolina, Columbia, SC 29212 USA; 5 School of Public Health, Kunming Medical University, Kunming, 650021 China; 6 School of International Education, Kunming Medical University, Kunming, 650021 China

**Keywords:** Mental health, SCL-90-R, Norms, Undergraduate students, Chinese version

## Abstract

**Background:**

Despite widespread application of the Symptom Check-List-90-R (SCL-90-R) for Chinese undergraduate students, there are no appropriate norms for them. The aim of this study is to provide norms for the Chinese version of the tool for undergraduate students using a large and representative sample**.**

**Methods:**

Four thousand eight hundred sixty students completed the scale of SCL-90. The mean scores obtained in the present study were compared with mean scores from previous normative samples.

**Results:**

The mean scores for nine subscales of the SCL-90-R ranged from (1.36 ± 0.46) ~ (1.77 ± 0.63) and the mean (standard deviation) *Global Severity Index* (*GSI*) was 1.50 (0.49). Relative to previous normative studies, the findings suggested that Chinese undergraduate students’ self-reported mental health symptoms decreased in interpersonal sensitivity, depression, hostility, and paranoid ideation subscales.

**Conclusion:**

It is necessary to revise the norms of the Chinese version of the SCL-90-R for undergraduate students.

## Background

The Symptom Check-List-90-R (SCL-90-R) is an extensively applied and well-known self-report scale that assesses a broad range of issues from mental health to psychopathological symptoms [1–3]. The SCL-90-R distinguishes patients with psychological problems from healthy people [4]. It was introduced into China by Wang [5] for studying people with psychiatric symptoms, and then used to measure the mental health of general adults and set norms for them in a study by Jin et al. [6] Subsequently, the scale has been widely used to evaluate the mental health of the general population in China, including students, teachers, workers, soldiers, nurses, doctors, and community residences, as well as psychiatric patients. The SCL-90-R has been used to measure the mental health and psychiatric symptoms of individuals as well as the overall evaluation and comparison of groups [7].

In the study by Jin [6] 1388 adults aged 18 ~ 60 years were surveyed in 13 regions of China, including 724 males and 664 females, incorporating people from all kinds of occupational and cultural groups but reporting no previous or current diagnosis of mental illness. It was found that scores on the nine most widely used subscales were similar, with mean scores ranging from 1.23 ~ 1.65. The sample participants were also divided into four age groups (18 ~ 29, 30 ~ 39, 40 ~ 49, 50 ~ 60 years) and the mean scores on the nine subscales were compared. The highest scores on a large number of subscales appeared in those aged 18 ~ 29 years, with scores on the interpersonal sensitivity, paranoid ideation, and psychoticism subscales being significantly higher than other age groups.

Over the past 30 years, much research on the mental health of the general population was evaluated with the SCL-90-R. It is the most commonly used scale to measure mental health in universities in China [8, 9]. Notably, more than half of the studies on the mental health status of Chinese undergraduate students have used the youth-group (age 18 ~ 29 years) norms from Jin’s study for normative comparisons [10].

Despite the frequent use of the 1986 norms, a number of studies have highlighted weaknesses of those norms. A central criticism has been that the norms are obsolete and non-representative. For example, the norms are based on a sample of adults aged 18 ~ 60 years and therefore do not represent the norms of undergraduate students in general since the age range for Chinese undergraduate students is 17 ~ 25 years. Also, the participants, including the youth-group sample, came from all kinds of occupational and cultural groups, and are therefore very different from current undergraduate students. Thus, the sample was not representative of undergraduate students. Finally, the norms have not been updated in 40 years. Given the substantial political, economic, and cultural changes that have occurred in Chinese society since 1986, the nature and prevalence of mental health problems have likely changed as well.

Multiple researchers have suggested that the norms should target undergraduate students to meet the current needs of researchers and practitioners. As a result, Zhang et al. [11], Tang et al. [12], Huang et al. [13], Zhong et al. [7], and Yang [14] successively established their own normative samples through a meta-analysis or re-analysis of Jin’s data. These norms were more recent and, although the studies focused on college students, were based on secondary data derived from the existing literature. Zhang et al. [11] chose five articles from published documents and formed a sample of 4141 college students from different schools and majors in the Shanghai, Hubei, Gansu, Guangzhou, Henan, and Anhui provinces in China. Based on the SCL-90-R test results reported in the five articles, they developed means and standard deviations for each subscale of the SCL-90-R via a meta-analysis. Tang et al. [12] compiled a sample of 23,891 college students from 169 articles by searching the main professional journals published from 1986 ~ 1999. They used the data from each study to calculate the scores for their norms. Huang et al. [13] developed norms by searching the Chinese Journal Full Text Database, Chinese dissertation database (CDMD), and VIP databases published from 1978 ~ 2008 and combined 232 studies to arrive at a sample of 263,775 college students from 24 provinces. Zhong et al. [7] integrated data from five published articles between 2002 and 2007 by searching the Chinese Journal Full Text Database. Finally, Yang [14] selected 49 published articles from 2000 ~ 2008 by searching the Chinese Journal Full Text Database. All of these researchers, therefore, re-analyzed information from existing literature as raw data and recalculated the means and standard deviations to develop their norms.

In response to such methods of developing norms, Zuo et al. [15] reported that these results were unacceptable and they simulated the processes of developing synthesized norms and applied a homogeneity test, subgroup analysis, regression analysis, and assessed publication bias to evaluate the results. The following four findings were obtained: (1) the hypothesis of homogeneity of somatization, anxiety, hostility, and phobic anxiety of the SCL-90-R was rejected and the other subscales retained homogeneity assumptions, but their *p*-values were close to 0.05; (2) the results of the meta-analysis also rejected the homogeneity hypothesis; (3) the sub-group analysis and regression analysis could not explain the source of heterogeneity; and (4) a test of publication bias revealed no publication bias in the extant literature. Xin et al. [10] also argued that these results were unacceptable because of the limitations of a meta-analysis itself. These limitations included the notion that the meta-analysis method (1) is too dependent on results that have been published (versus those results that have not been published), (2) may ignore important individual differences among studies in the integration of the findings, and (3) is easily influenced by the quality and quantity of literature. Moreover, there is also the problem of the varying dates of the studies included in the meta-analysis. Many meta-analyses have found that significant correlation exists between the date of data collection and the research results. Together, these findings suggest problems with the validity of synthesized SCL-90-R norms, which are established on the basis of secondary data collected from the literature.

Most researchers have claimed that it is necessary to establish norms by directly collecting firsthand information using questionnaires or surveys with undergraduate students. Several researchers [16–20] have revised the SCL-90-R norms for undergraduate students by acquiring direct data. However, all these studies only sampled one province or district for their surveys. Thus, their norms are only suitable for the particular provinces or districts sampled and not for China as a whole. Tong [21] sampled 1890 adults aged 17 to 84 years from 21 provinces in China and analyzed the scores from all participants. Unfortunately, the scores of young people (including undergraduate students) were not analyzed separately from the adults. Therefore, norms for young people or undergraduate students could not be derived from this study since the original study by Jin found significant differences between age groups.

Furthermore, the *Global Severity Index* (*GSI*) value was reported in only a few studies on norms formulation in China. The SCL-90-R has been widely used to measure the symptoms of patients and the general population; however, GSI values differ by type of population with asymptomatic, mild, moderate, and severe symptoms [22].

The aim of the present study is to update and report norms for SCL-90-R and its subscales based on a representative, normative sample appropriate for use with Chinese undergraduate students. The findings of this study would add to the extant literature by clarifying the usefulness of a widely used measure and updated normative standard for assessing the current psychological problems of students in China and provide a reference for Chinese practitioners to conduct mental health assessments. The GSI value was also calculated in the present study to facilitate a comparison of Chinese and international research, as well as a reference for international researchers. Based on this sample, we have evaluated the psychometric properties and applicability [9], which showed that the scale for university students was applicable. Norms would be set for undergraduate students in this paper.

## Methods

### Sample

A multistage, stratified cluster sampling strategy was used to recruit 4860 undergraduate studentsfrom the northeast, northwest, west, east, south, and central regions of China, 6 provinces in 2014. The participants ranged in age from 17 to 25. Xin et al. [10] compared the changes in the mental health of Chinese undergraduate students from 1986 ~ 2010 and found that the differences were mainly in the students’ grade (freshman, sophomore, junior, senior), gender, type of university (key, good, ordinary), and area of residence (urban, rural). Therefore, we randomly selected an equal number of students in the four grades, stratifying by the type of university (key, good, ordinary) using a ratio of 2:5:3 to match that of the population of undergraduate students in China [23].

### Procedure

Firstly, researchers obtained consent and support from the counselors for each class and explained the aims of the study. Secondly, researchers stressed that the survey results would be kept confidential and that the student’s names would be anonymous. In addition, the researchers informed the students about the purpose and importance of the study. Following the explanation of the study, the researchers obtained the students’ written consent before participation. Thirdly, research assistants introduced themselves using a standardized script. Finally, students filled in the questionnaire in their classroom. Participants had 45 min to complete the scale and returned it immediately to the research assistants when they finished.

### Instrument

The SCL-90-R [2, 24] contains 9 subscales: Somatization (SOM), Obsessive-Compulsive (O-C), Interpersonal Sensitivity (I-S), Depression (DEP), Anxiety (ANX), Hostility (HOS), Phobic Anxiety (PHOB), Paranoid Ideation (PAR), and Psychoticism (PSY), and the nine frequently used subscales provide symptom profiles. It is a five-point Likert scale ranging from 1 to 5; “1: not at all”, “2: a little bit”, “3: moderately”, “4: quite a bit” and “5: extremely.” Thus, a higher score reflects a higher frequency or intensity of symptoms. The *Global Severity Index* (*GSI*) is equal to the average of all nine subscale scores.

### Statistical analysis

JASP-0.12.2 and R 3.6.1 were used to analyze the data. An analysis of missing data led to the exclusion of 404 participants as they showed more than the tolerated amount of missing data (tolerated < 3 items in the SCL-90-R scale, < 6 items in total). The percentage of missing values of each item ranged between 0.2 and 0.5% and were not assigned randomly (Little MCAR-Test: χ2 = 11,552.3, df = 7180, *p* < .001). Therefore, they were replaced using a multiple imputation technique to avoid selection bias.

Chi-squared tests were used to compare socio-demographic characteristics (gender, type of university, grade). Bayesian independent sample T-tests using the Jeffreys-Zellener-Siow prior were used to compare differences between male and female students and between those living in urban and rural areas. A Bayesian one-sample T-test was used to compare our results with those of the youth-group reported by Jin et al [6] Cronbach’s α, McDonald’s ω, Spearman-Brown Split-half reliability, and intra-class correlation coefficients (ICC) were used to determine the reliability of the SCL-90-R and its subscales. A two-way random model, absolute agreement type, and 95% confidence interval for the ICC were used. Reliability is generally considered good when α and ω exceeds 0.70 [25–27] and the ICC is greater than 0.80 [28].

## Results

### Representativeness of sample

We received 4456 valid questionnaires. Table 1 shows the demographic characteristics of the study sample and a comparison with the undergraduate population of China. The percentages of males and females were 52.6 and 46.5% in the present study, respectively, which was similar to the actual percentages in undergraduate students in China of 47.5 and 52.5%. Other demographic variables in the sample were also similar to the general population in terms of type of university, grade, and area of residence. We can therefore infer that the study sample is representative of college students in China.

**Table 1:** Demographic characteristics of undergraduate students

		Sample		Population	*p*-value
		*n*	%	%	
Gender	Male	2344	52.6	47.5	0.530
	Female	2077	46.6	52.5	
	Unknown	35	0.8		
Type of university	Excellent	942	21.1	20.0	0.996
	Good	2217	50.0	50.0	
	Ordinary	1297	29.1	30.0	
Grade	Freshman	1336	30.0	25.6	0.994
	Sophomore	1224	27.5	25.6	
	Junior	1002	22.5	25.5	
	Senior	894	20.1	23.3	
Area of residence	Urban	2236	50.2	47.5	0.70
	Rural	2123	47.6	52.5	
	Unknown	97	2.2		

### Reliability analysis

Table 2 shows the reliability statistics for the 9 subscales of the SCL-90 plus the overall value. The results showed that the total scale and 9 subscales all displayed high internal consistency.

**Table 2:** Reliability analyses for various subscales of the SCL-90

	Cronbach’s α	McDonald’s ω	Spearman-Brown Split-half reliability	Intra-class correlation coefficient
SOM	0.88	0.89	0.85	0.88
O-C	0.86	0.87	0.85	0.86
I-S	0.86	0.86	0.85	0.86
DEP	0.91	0.91	0.90	0.91
ANX	0.88	0.89	0.87	0.88
HOS	0.80	0.81	0.84	0.80
PHOB	0.81	0.81	0.82	0.81
PAR	0.78	0.79	0.79	0.80
PSY	0.85	0.85	0.83	0.85
SCL-90	0.98	0.98	0.95	0.98

### Norms for undergraduate students

The norms of the SCL-90-R subscales, GSI, and gender-specific norms for undergraduate students in China are shown in Table 3. A comparison of the norms of the subscales and GSI values between males and females showed that all the posterior medians were negative indicating that males had lower scores on average than females All Bayes factors were greater than 100 except for the HOS subscale which was 54.9. This shows that the mean values of all subscales, except for hostility, in gender were over 100 times more likely to be unequal than equal.

**Table 3:** Chinese national and gender-specific norms for the SCL-90 subscales

	Gender			BF_10_	Posterior median	95% CI
	All	Male	Female			
SOM	1.36 ± 0.46	1.33 ± 0.45	1.40 ± 0.47	8500	−0.15	− 0.21, − 0.09
O-C	1.77 ± 0.63	1.68 ± 0.62	1.88 ± 0.63	8.34 × 10^21^	− 0.31	− 0.37, − 0.26
I-S	1.60 ± 0.60	1.53 ± 0.59	1.67 ± 0.62	1.99 × 10^10^	−0.22	− 0.28, − 0.16
DEP	1.52 ± 0.58	1.46 ± 0.56	1.60 ± 0.60	6.78 × 10^12^	−0.25	−0.30, − 0.19
ANX	1.49 ± 0.57	1.42 ± 0.54	1.58 ± 0.59	3.54 × 10^17^	−0.28	−0.34, − 0.22
HOS	1.46 ± 0.55	1.43 ± 0.55	1.49 ± 0.55	54.94	−0.12	−0.18, − 0.06
PHOB	1.36 ± 0.51	1.30 ± 0.49	1.43 ± 0.53	6.81 × 10^13^	−0.25	−0.31, − 0.19
PAR	1.46 ± 0.53	1.44 ± 0.53	1.50 ± 0.53	41.22	−0.11	−0.17, − 0.05
PSY	1.44 ± 0.52	1.41 ± 0.53	1.47 ± 0.50	165.3	−0.12	−0.18, − 0.07
GSI	1.50 ± 0.49	1.44 ± 0.48	1.56 ± 0.49	1.03 × 10^12^	−0.24	−0.30, − 0.18

Note: *BF10* Bayes Factor, *CI* Confidence Interval

Table 4 compares the norms between urban and rural undergraduate students. All posterior medians were negative indicating that students from urban had lower scores on average than those from rural. Bayes factors of interpersonal sensitivity, phobic anxiety, and psychoticism subscales were greater than 100, obsessive-compulsion was 8.15, and the other subscales were all less than 3. The GSI was 16.4. This indicates that the mean values of interpersonal sensitivity, phobic anxiety, and psychoticism subscales between urban and rural students were over 100 times more likely to be unequal than equal, the mean value of GSI in the two groups was 16.4 times more likely to be unequal than equal, the mean of the obsessive-compulsive subscale in the two groups were 8.15 times more likely to be unequal than equal, and for the other subscales, the likelihood was less than 3.

**Table 4:** Chinese national and residential-specific norms for the SCL-90 subscales

	Urban	Rural	BF_10_	Posterior median	95% CI
SOM	1.35 ± 0.47	1.37 ± 0.46	0.06	−0.03	−0.09, 0.03
O-C	1.74 ± 0.64	1.80 ± 0.62	8.15	−0.10	−0.16, − 0.04
I-S	1.56 ± 0.60	1.63 ± 0.61	177.2	−0.13	−0.19, − 0.07
DEP	1.50 ± 0.59	1.54 ± 0.58	0.41	−0.07	−0.13, − 0.01
ANX	1.47 ± 0.56	1.52 ± 0.58	2.08	−0.09	−0.15, − 0.03
HOS	1.44 ± 0.60	1.47 ± 0.53	0.43	−0.07	−0.13, − 0.01
PHOB	1.32 ± 0.48	1.41 ± 0.54	501,028	−0.17	−0.23, − 0.12
PAR	1.45 ± 0.54	1.48 ± 0.52	0.15	−0.05	−0.11, 0.01
PSY	1.40 ± 0.51	1.47 ± 0.52	3227	−0.15	−0.20, − 0.09
GSI	1.47 ± 0.48	1.52 ± 0.49	16.4	−0.11	−0.17, − 0.05

Note: *BF10* Bayes Factor, *CI* Confidence Interval

### Norms of the SCL-90-R in the present study and comparison with previous norms in China

Table 5 shows a comparison of norms between this study and 9 previous studies conducted in China. The first 4 studies were meta-analyses and the remaining 5 studies were cross-sectional surveys conducted in a single province of China. All 9 studies, except for the one by Jin et al. [6] which included adults, sampled undergraduate or college students. Except for Jin’s study, and the phobic anxiety subscale in the study by Wang [20], all the SCL-90-R subscale scores in the present study were lower than those reported in previous studies. The scores of the present study were closest to the scores of Jin et al [6] From the Bayesian one-sample T-tests, comparing the present study with the one by Jin et al. [6] Bayes factors for somatization was 0.63 and for phobic ideation was 83. For all other subscales, the Bayes factors were greater than 100 (BF_10_ of O-C was 3.59 × 10^14^, BF_10_ of I-S was 9.64 × 10^66^, BF_10_ of DEP was 50,651.67, BF_10_ of ANX was 1.93 × 10^14^, BF_10_ of HOS was 41,590.60, BF_10_ of PHOB was 83.21, BF_10_ of PAR was 3.35 × 10^9^, BF_10_ of PSY was 2.23 × 10^19^). This showed that the scores of all but two subscales between the present study and Jin’s study were more than 100 times more likely to be unequal than equal.

**Table 5:** Comparison of norms between the present study and previous studies (mean ± standard deviation)

	Zhang Z	Tang Q	Huang Y	Zhong W	Jin H^a^	Ji J	Wang Y	Wang T	Luo W	Yu Y
Sample size	4141	23,891	263,775	9941	781	547	910	1350	1330	4456
Area	China	China	China	China	China	Shanghai	Shanxi	Jiangsu	Zhejiang	China
Method	Meta-analysis	Meta-analysis	Meta-analysis	Meta-analysis	Survey	Survey	Survey	Survey	Survey	Survey
Year	1998	1999	2009	2009	1986	1990	2003	2007	2009	2014
SOM	1.45 ± 0.49	1.44 ± 0.51	1.39 ± 0.47	1.45 ± 0.49	1.34 ± 0.45	1.41 ± 0.42	1.44 ± 0.43	1.37 ± 0.40	1.48 ± 0.48	1.36 ± 0.46
O-C	1.98 ± 0.64	1.92 ± 0.64	1.87 ± 0.62	1.98 ± 0.63	1.69 ± 0.61	1.99 ± 0.68	2.01 ± 0.62	1.92 ± 0.56	1.82 ± 0.55	1.77 ± 0.63
I-S	1.98 ± 0.74	1.85 ± 0.64	1.79 ± 0.59	1.88 ± 0.63	1.76 ± 0.67	2.02 ± 0.71	1.89 ± 0.63	1.75 ± 0.55	1.69 ± 0.56	1.60 ± 0.60
DEP	1.83 ± 0.65	1.76 ± 0.64	1.67 ± 0.62	1.74 ± 0.62	1.57 ± 0.61	1.83 ± 0.68	1.77 ± 0.60	1.65 ± 0.54	1.65 ± 0.57	1.52 ± 0.58
ANX	1.64 ± 0.59	1.59 ± 0.57	1.55 ± 0.54	1.61 ± 0.55	1.42 ± 0.43	1.64 ± 0.57	1.64 ± 0.54	1.56 ± 0.48	1.57 ± 0.53	1.49 ± 0.57
HOS	1.77 ± 0.68	1.68 ± 0.65	1.58 ± 0.59	1.61 ± 0.62	1.50 ± 0.57	1.75 ± 0.68	1.64 ± 0.61	1.57 ± 0.53	1.58 ± 0.57	1.46 ± 0.55
PHOB	1.46 ± 0.53	1.42 ± 0.51	1.40 ± 0.51	1.38 ± 0.49	1.33 ± 0.47	1.44 ± 0.50	1.43 ± 0.45	1.31 ± 0.36	1.46 ± 0.51	1.36 ± 0.51
PAR	1.85 ± 0.69	1.78 ± 0.65	1.63 ± 0.57	1.72 ± 0.65	1.52 ± 0.60	1.89 ± 0.68	1.75 ± 0.56	1.59 ± 0.50	1.60 ± 0.54	1.46 ± 0.53
PSY	1.63 ± 0.54	1.58 ± 0.54	1.50 ± 0.51	1.59 ± 0.54	1.36 ± 0.47	1.63 ± 0.53	1.61 ± 0.52	1.51 ± 0.44	1.50 ± 0.51	1.44 ± 0.52

^a^Sample included adults

## Discussion

Although the SCL-90-R has been extensively applied in the general population, especially for college students, its use has been controversial from the beginning [29]. One of the most obvious controversies concerns the norm of the SCL-90. The SCL-90 is a standard reference test in the use of self-evaluation of symptoms of patients. The so-called standard referenced test refers to a test that directly interprets the test results in certain behavioral areas according to specific behavioral standards. The interpretation of scores is based on the standard set by the examinees before the test implementation instead of the norm.

Norm reference test means comparing the measurement result with the norm in order to determine the relative position of the subjects in the group. Since Jin et al. [6] used SCL-90-R to evaluate the mental health of the general population, which includes those with no mental health symptoms and therefore provides the norm of ordinary people, the majority of research studies in China have mainly focused on the subjects of measuring relative position to the norm, so the measurement properties of SCL90-R have changed and became the norm-referenced test and diagnostic tool. Therefore, Gao et al. [30] suggested that researchers pay more attention to the scope and properties of the scale when using it for mental health assessment. The norm of the SCL-90-R for Chinese ordinary people has been established for more than 30 years and there has been no authoritative revision during this period. Over the past 30 years, great changes have taken place in Chinese society which has affected people’s mental health. It is therefore important to update the norms. Furthermore, specific norms for undergraduate students is necessary. Chen et al. [31] proposed to reformulate the norm of the SCL-90-R according to different occupations and age groups. Undergraduate students have their own characteristics and they differ from other adults because of their identity as students.

The sample used to define the norms must be representative of the population of interest. Chinese norms must focus on the whole country instead of just one particular province or region. Table 1 showed that the sample of the present study was representative of undergraduate students in China. Furthermore, Table 5 showed that our sample was the most representative sample among 9 other studies because we sampled from all regions of the country and used primary data.

**Fig. 1:**
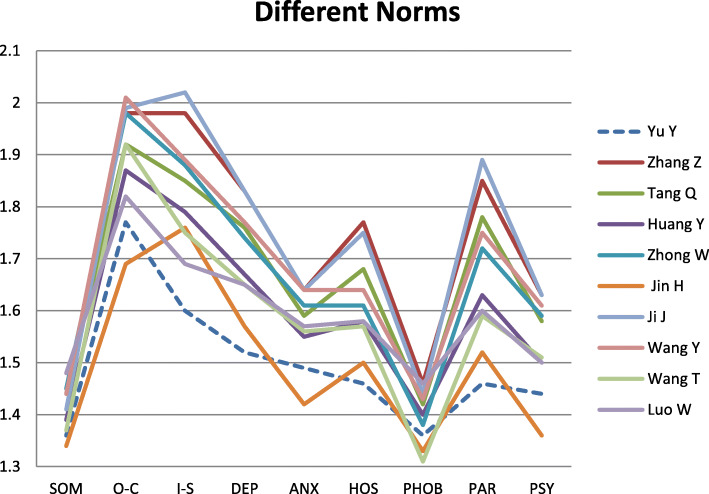
Comparison of norms between the present study and previous studies

Figure 1 shows a comparison of the norms between all 10 studies. Results of the present study were cloest to those by Jin and the scores of both studies were lower than the other studies. The study of Jin was also similar to the present study in terms of methodology. Both studies recruited participants from the whole country and used primary data instead of secondary data.

Our results were consistent with a study of Xin et al. [10] who examined the mental health trends of undergraduate students over time. They found that scores for paranoid ideation, interpersonal sensitivity, depression, and hostility subscales had decreased from 1986 to 2010. From Fig. 1, we can see that the scores for these four subscales from our study were lower than the other older studies.

In summary, considering the representative sample, sound study methods, and the evidence from other studies, the results of this present study can be used as norms for undergraduate students in China.

From Table 3, all Bayes factors were greater than 100 except for the hostility subscale which was 54.9. This indicates that there was strong evidence of inequality in almost all subscales between male and female students. We therefore show gender-specific norms for the 9 subscales in addition to the overall scores. We also present area-specific norms for students in Table 4.

The new norms developed in the present study will provide a reference to assess undergraduate students’ mental health status correctly for researchers and practitioners. The Global Severity Index (GSI) was provided which was lacking in most other Chinese studies. Gender-specific and area-specific normswere also provided to meet the specific needs of researchers.

The scores of the present study can be used as new norms for the following reasons. First, we acquired primary data using a cross-sectional survey. Secondly, the sample was representative of undergraduate students in China by recruiting a large number of participants randomly sampled from all regions of the country. Third, the present results were validated as evidenced by consistent results with other studies [10]. Furthermore, a Bayesian analysis was used to obtain a more rigorous result. This method is more suitable than using classical null hypothesis significance testing because, in large samples, small deviations can produce statistically significant differences, which may have no practical importance.

Although this study used primary data to establish norms for undergraduate students in China, it has some limitations. First, the SCL-90-R is originally a standard reference test rather than a norm reference test. Whether it is appropriate to translate it into a norm reference to evaluate the mental health status of the general population remains to be further studied. Second, there were no non-undergraduate students to use as a control group. Third, we did not compare the Global Severity Index with other studies because it was rarely reported in other studies in China, although it is an important indicator. Despite these limitations, this study adds to the literature by establishing new norms of SCL-90-R for undergraduate students in China.

## Conclusion

The present study has made some important contributions in revising the norms for undergraduate students in China by analysing a large, representative sample using a scientific method. Compared to previous normative studies, the findings suggested that Chinese undergraduate students in this study reported lower symptoms in interpersonal sensitivity, depression, hostility, and paranoid ideation subscales.

## Data Availability

Not Applicable.

## Abbreviations

ANX: Anxiety
DEP: Depression
HOS: Hostility
I-S: Interpersonal Sensitivity
O-C: Obsessive-Compulsive
PAR: Paranoid Ideation
PHOB: Phobic Anxiety
PSY: Psychoticism
SOM: Somatization
SCL-90-R: Symptom Check List 90
GSI: Global Severity Index
